# Molecular Genetics of Renal Cell Tumors: A Practical Diagnostic Approach

**DOI:** 10.3390/cancers12010085

**Published:** 2019-12-30

**Authors:** Reza Alaghehbandan, Delia Perez Montiel, Ana Silvia Luis, Ondrej Hes

**Affiliations:** 1 Department of Pathology, Faculty of Medicine, University of British Columbia, Royal Columbian Hospital, Vancouver, BC V3E 0G9, Canada; reza.alagh@gmail.com; 2 Department of Pathology, Institute Nacional de Cancerologia, INCAN, Mexico DF 14080, Mexico; madeliapmg@yahoo.com.mx; 3 Department of Pathology, Centro Hospitalar de Vila Nova de Gaia-Espinho, Vila Nova de Gaia, Cancer Biology and Epigenetics Group (CBEG), IPO Porto Research Center (CI-IPOP), Portuguese Oncology Institute of Porto (IPO Porto) & Porto Comprehensive Cancer Center (P.CCC), 4200-072 Porto, Portugal; anasilvialuis@gmail.com; 4 Department of Microscopy, Institute of Biomedical Sciences Abel Salazar, University of Porto (ICBAS-UP), 4200-072 Porto, Portugal; 5 Department of Pathology, Charles University in Prague, Faculty of Medicine in Plzen, 304 60 Pilsen, Czech Republic

**Keywords:** kidney, renal cell carcinoma, molecular genetic features, practical approach, review

## Abstract

Renal epithelial cell tumors are composed of a heterogeneous group of tumors with variable morphologic, immunohistochemical, and molecular features. A “histo-molecular” approach is now an integral part of defining renal tumors, aiming to be clinically and therapeutically pertinent. Most renal epithelial tumors including the new and emerging entities have distinct molecular and genetic features which can be detected using various methods. Most renal epithelial tumors can be diagnosed easily based on pure histologic findings with or without immunohistochemical examination. Furthermore, molecular-genetic testing can be utilized to assist in arriving at an accurate diagnosis. In this review, we presented the most current knowledge concerning molecular-genetic aspects of renal epithelial neoplasms, which potentially can be used in daily diagnostic practice.

## 1. Introduction

Renal cell tumors are one of the most extensively studied human neoplasms. A number of morphologic, immunohistochemical and molecular genetic features were described during the last 20 years, which have also led to recognition of new entities, expanding our knowledge and understanding of renal tumors.

“The Heidelberg classification of renal cell tumors” published in 1997 was the first classification integrating molecular genetic features as one of the diagnostic tools applicable to renal cell tumors [1]. This classification was further corroborated by the so-called UICC Rochester Classification [2], which later evolved through the 2004 World Health Organization (WHO) tumor classification [3], 2012 Vancouver ISUP (International Society of Urologic Pathology) consensus conference [4], and most recently the 2016 WHO blue book [5]. This “histo-molecular” approach is now an integral part of defining renal tumors and the emerging entities, aiming to be clinically and therapeutically pertinent. A summary of genetic tests and routinely used immunohistochemical examinations in daily practice is shown in Table 1. There have been many studies describing and examining molecular genetic changes of renal tumors. All these studies have shown that the molecular genetic changes are remarkably heterogeneous across the whole spectrum of renal cell carcinomas (RCCs) and other tumors and that molecular-genetic analysis cannot be used as a universal diagnostic tool.

In this review, we present the most current knowledge concerning molecular-genetic aspects of renal epithelial neoplasms, which potentially can be used in daily diagnostic practice. It is important to note that ISUP recommendations for molecular genetic testing of renal cell tumors will be published in the near future.

## 2. Clear Cell Renal Cell Carcinoma (CCRCC)

Clear cell renal cell carcinoma (CCRCC), the most common RCC, is typically composed of cells with clear cytoplasm and with a rich fine capillary network [5]. CCRCCs can also exhibit with eosinophilic cytoplasm and marked cellular pleomorphism. CCRCC cases mimicking clear cell papillary RCC are not infrequently found [6,7].

Chromosome 3p deletion has been described in CCRCC since the 80s [8], and was recognized as a characteristic genetic feature of this tumor in the Heidelberg classification [1], present in more than 90% of cases [9,10]. In parallel, *VHL* gene located on chromosome 3p was described as the most frequently mutated gene (50–75%) in CCRCC, and later found to be silenced by promoter methylation in 5–20% of cases [9,10]. Thus, the most frequent genetic alteration in CCRCC involves chromosome 3p deletion, *VHL* mutation and/or *VHL* promoter methylation, leading to *VHL* inactivation, an early and crucial event in sporadic CCRCC and in the familial cancer syndrome von Hippel–Lindau disease [9,11].

In addition to *VHL* located on chromosome 3p, there are other genes such the component of the SWI/SNF chromatin remodeling complex PBRM1 (26–33%), the histone modifying enzymes SETD2 (4–12%) and BAP1 (10%) being frequently reported in CCRCC [12,13,14].

Application in routine practice:

The presence of *VHL* mutation, chromosome 3p deletion or *VHL* promoter methylation is considered useful for the confirmation of CCRCC diagnosis in difficult cases (see following sections).

## 3. Multilocular Cystic Renal Cell Neoplasm of Low Malignant Potential (MCRCNLMP)

Multilocular cystic renal cell neoplasm of low malignant potential (MCRCNLMP) is a rare (<1%) renal tumor with an excellent prognosis, without recurrence or metastases described for bona fide cases [5]. Careful macroscopic evaluation and sampling are pivotal for the diagnosis, as the presence of solid nodules and/or cell clusters with expansive growth warrants a diagnosis of multicystic CCRCC [5]. MCRCNLMP, a low-grade tumor, had been considered a variant of CCRCC due to morphological similarities and analogous genetic features [15,16,17]. The most frequent genetic alterations in MCRCNLMP are identical to chromosome 3p deletion in 74% (14/19) of cases [17] and *VHL* mutation in 25% (3/12) of cases [18].

Interestingly, *KRAS* mutation was not found in small cohort of 12 MCRCNLMP cases, contrarily to codon 12 or codon 13 mutations identified in 12 CCRCC cases [19,20]. Of note, other studies that failed to identify *KRAS* mutation in CCRCC sequenced only codon 12 [21], codon 2 [22] or codons 1 and 2 [23], or used distinct methodology [24,25], which might have contributed to the conflicting results.

Application in routine practice:

Similar to CCRCC, chromosome 3p deletion and *VHL* mutation might be found in MCRCNLMP, but no specific genetic alterations have so far been identified. Careful macroscopic and microscopic evaluation is the gold standard for diagnosis.

## 4. Papillary Renal Cell Carcinoma (PRCC)

Papillary renal cell carcinoma (PRCC) is the second most common type of RCC, traditionally referred as tumor comprising of 15% of all RCCs [26]. According to the latest classification systems (WHO 2004, Vancouver ISUP Classification), it is classified into type 1 and type 2 PRCCs, which is also currently being used in the latest WHO 2016 classification. While PRCC type 1 seems to be a distinct and compact histo-molecular entity, the so-called type 2 appears to be, rather, composed of a group of tumors sharing papillary/tubulopapillary architecture with different molecular and genetic features [27]. In addition, there have recently been a number of subtypes/variants of papillary renal tumors (i.e., fumarate hydratase (FH)-deficient RCC, oncocytic PRCC), expanding the PRCC spectrum [27].

### 4.1. Type 1 Papillary RCC

The morphology of PRCC type 1 is well defined and in most cases would suffice for an accurate diagnosis in routine practice [5]. These tumors also have a distinct immunohistochemical profile, which can be further utilized in addition to basic hematoxylin and eosin (H&E) staining in the diagnostic workup [5].

The CNV (copy-number variation) pattern is relatively constant demonstrating polysomy or trisomy of chromosomes 7 or 17 as the most frequently referred changes. However, gains of chromosomes 3, 12, 16, and 20 (and less frequently gains of chromosomes 2, 4, 5, 6, 8, 13, and 18) have also been noted in these tumors. Of note, chromosomal losses have also been reported (chromosomes 1, 2, 4, 5, 7, 8, 9, 10, 11, 14, 15, 16, 18, 19, 20, 21, and 22) [28].

While mutations of *MET* are rarely referred for sporadic type 1 PRCC, it is commonly associated with hereditary papillary RCC syndrome. It should be noted that tumors occurring within hereditary papillary RCC syndrome are multiple otherwise typical PRCCs type 1 [29,30,31].

### 4.2. Type 2 Papillary RCC

“Type 2” papillary RCC is considered a controversial entity and currently by most authors deemed rather to represent multiple specific papillary renal neoplasms. Although gains of chromosomes 7 and 17 were reported to be the most frequently listed CNV changes for this subtype, the recent literature show that trisomy/polysomy 7/17 is not commonly associated with type 2 PRCC [28]. Based on a systematic review published recently, gains of chromosomes 12, 16 and 20 are also frequently reported in papillary RCC “Type 2” [28].

There are several genetic-based studies supporting the notion that the so-called “Type 2” PRCC is rather a group of tumors. Such tumors showed *CDKN2A* silencing, *SETD2* mutations, and increased expression of the NRF2 antioxidant response element pathway [27]. Of note, FH-deficient RCCs, a high-grade PRCC which was previously categorized as PRCC “Type 2”, have already been reclassified from Type 2 PRCC, owing to recent molecular and genetic studies on Type 2 PRCCs [27].

### 4.3. Oncocytic Papillary RCC/Papillary Renal Cell Neoplasm with Reverse Polarity

Oncocytic papillary RCC, the “third” variant/subtype of papillary RCC included in the WHO 2016 blue book [32,33,34,35], is a poorly understood papillary RCC entity composed of oncocytic neoplastic cells [26,36]. CNV pattern in these tumors is highly variable with at least 3 patterns being reported: (1) gains of chromosomes 7 and 17 [33,34,35], (2) gains of chromosomes 3 and 11, and (3) loss of chromosome Y in male patients as well as losses of chromosomes 1, 4, 14 and loss of chromosome X. Some these tumors have shown to have a copy number pattern identical to renal oncocytoma: disomic status of chromosomes 7 and 17, some with deletion of chromosome 14, deletion of 1p (locus 1p36) [37]. Saleeb et al. considered oncocytic PRCC as so-called “type 4” papillary RCC (oncocytic low-grade), [38]. Al-Obaidy et al. have proposed the term papillary renal cell neoplasm with reverse polarity at last for a part of the spectrum of oncocytic RCC with papillary architecture [39]. Interestingly, this tumor is characterized by frequent *KRAS* mutations [40].

### 4.4. Papillary RCC NOS: Other Variants

A number of unusual papillary RCC variants have recently been described such as solid, mucin secreting, biphasic squamoid, and Warthin-like, which potentially can create diagnostic challenges in routine practice [41,42,43,44,45,46,47]. It should be noted that all of these variants are defined mostly using morphologic features and that their molecular-genetic features are widely varied, as generally observed in papillary RCCs.

Application in routine practice:

PRCC type 1 is a distinct entity, demonstrating typical CNV of gain of 7, 17 and loss of the Y chromosome in male patients. PRCC type 2 is rather composed of a group of tumors without a consistent CNV pattern. Considering highly variable CNV patterns among PRCCs in general (except for type 1 PRCC), it is almost impossible to diagnose PRCC based on CNV or based on another molecular genetic methods only. In high grade papillary renal tumors, FH-deficient RCC should always be considered and ruled out using a combination of immunohistochemistry and *FH* mutation/LOH (loss of heterozygosity) analysis.

## 5. Chromophobe Renal Cell Carcinoma (ChRCC)

Molecular-genetic testing will not be required to make a diagnosis of typical chromophobe RCC (ChRCC) (classic or eosinophilic variants). In addition to classic and eosinophilic ChRCCs, there are several other variants which have been described in the literature including pigmented microcystic adenomatoid, multicystic variant, [48,49,50,51] ChRCC with neuroendocrine differentiation, [52,53,54,55,56] and renal oncocytoma-like variant [57]. With the exception of ChRCC with neuroendocrine features, it seems that such variability has no influence on biological behavior. However, ChRCC with neuroendocrine features is a more aggressive variant [52].

CNV in ChRCC is rather variable and as such would be challenging to utilize in routine practice. ChRCC is usually associated with multiple chromosomal losses including chromosomes Y, 1, 2, 6, 10, 13, 17, 21 [58,59]. However, multiple chromosomal gains (chromosomes 4, 7, 15, 19, and 20), or even diploid pattern have been described in otherwise typical CHRCCs [60,61,62,63].

Testing germline mutations in the novel tumor suppressor gene *FLCN* (folliculin) can be used to support the diagnosis of Birt–Hogg–Dubé syndrome, which predisposes to the so-called “hybrid” oncocytic/chromophobe tumors.

Application in routine practice:

Molecular genetic ancillary tests are not useful for diagnosing ChRCC in daily practice. However, *FLCN* gene analysis can be useful in “hybrid” oncocytic/chromophobe tumors in suspected cases.

## 6. Oncocytoma

Renal oncocytoma (RO) can mostly be diagnosed based on morphology, while in difficult cases immunohistochemical examination can be further utilized. Molecular genetic tests are rarely used to diagnose RO. There are 3 basic genetic patterns in ROs: (1) loss of chromosome 1 (in whole or in part) and loss of chromosome Y, (2) rearrangements of 11q13 (mostly translocation t(5;11)(q35;q13)), chromosome 14 deletion, and (3) a normal karyotype [64,65,66,67,68,69].

These patterns have led some authors to propose two or three dominant subtypes of RO, however the clinical utility of such categorization remains unclear [69,70]. It has recently been recognized that CCND1 (cyclin D1) is located on the 11q13 locus. There are several studies that have attempted to sub-classify ROs according to the CCND1 status [69,70,71]. Nonetheless, all these proposals have shown no clinical usefulness and utility in daily routine and differential diagnostic practice. Similar to ChRCCs, the most commonly used test is the analysis of the *FLCN* gene in a similar setting, such as in “hybrid” oncocytic/chromophobe tumors.

Application in routine practice:

Similar to ChRCCs, molecular-genetic ancillary tests are not very useful for ROs in daily practice. However, *FLCN* gene analysis in suspected cases of “hybrid” oncocytic/chromophobe tumors is useful.

## 7. Clear Cell Papillary Renal Cell Carcinoma (CCPRCC)

The diagnosis of typical clear cell papillary renal cell carcinoma (CCPRCC) is mostly based on the morphology and immunohistochemical profile. In typical cases with characteristic morphology, diffuse CK7 positivity, and strong cup-shaped positivity with CANH 9, molecular genetic testing is not necessary for the diagnosis of CCPRCC [72,73,74]. However, there are some cases of CCPRCCs which show more complex morphologic features and substantial overlap with other RCCs (i.e., CCRCC). Because CCPRCC is an indolent neoplasm (with few extremely rare exceptions), an accurate diagnosis is crucial for further management. We believe that in such instances, further analysis of the *VHL* gene is the most useful and valuable step in arriving at the correct diagnosis in routine practice [75]. In fact, analysis of 3p25 loss and *VHL* gene alterations (mutations and methylation status) together with morphology and immunohistochemical profile would allow us to correctly diagnose almost all such cases [75].

It is important to emphasis that CCPRCC has been described in patients with von Hippel–Lindau syndrome. In such cases, *VHL* germline mutation is an obvious finding and can’t be helpful in differential diagnostic process.

Application in routine practice:

Majority of CCPRCC are diagnosed based on morphology and immunogistochemical profile. In challenging cases where the morphology and/or immunohistochemical profile are not typical of CCPRCC, genetic testing for *VHL* mutation/methylation and/or chromosome 3p loss are essential for rendering an accurate diagnosis of CCPRCC.

## 8. MiT Family Translocation-Associated Renal Cell Carcinoma

Renal tumors with *TFE3*, *TFEB*, and *MiTF* rearrangements are “classic” translocation-associated RCCs, being diagnosed based on a combination of morphologic, immunohistochemical, and molecular genetic analyses. RCC with *TFE3* rearrangements (Xp11.2) is the most common of all translocation-associated RCCs. Although morphologic features of translocation-associated RCCs are well described in the literature, recent studies have described morphologic variants associated with different fusion partners, which can in itself pose challenges to the diagnostic process. Some of these tumors are surprisingly similar in morphology to clear cell papillary RCC (*TFE3-NONO*). So far the following fusion partners for *TFE3* gene have been described: *ASPSCR1*, *PRCC*, *NONO*, *SFPQ*, *CLTC*, *PARP14*, *LUC7L3*, *KHSRP*, *DVL2*, *MED15*, *NEAT1*, *RBM10*, *KAT6A*, and *GRIPAP1* [76,77,78,79,80,81,82,83,84].

Although TFE3 translocation RCCs can show a diverse morphologic spectrum, certain morphologic features (i.e., high-grade cells with abundant clear/eosinophilic cytoplasm and papillary/nested architecture; psammomatous calcifications) can be suggestive of this entity. Immunohistochemical analysis may not be sufficient to confirm the diagnosis of TFE3 translocation RCC, and that in some cases further molecular genetic testing maybe indicated [85]. Fluorescence in situ hybridization (FISH) testing is usually used to confirm the diagnosis. It should be noted that in some fusion partners, FISH can produce false negative results [85,86]. Thus, NGS is more accurate, namely for cases, where fusion partner is beyond the reaches of probe or staying too close to *TFE3.*

**Figure 1:**
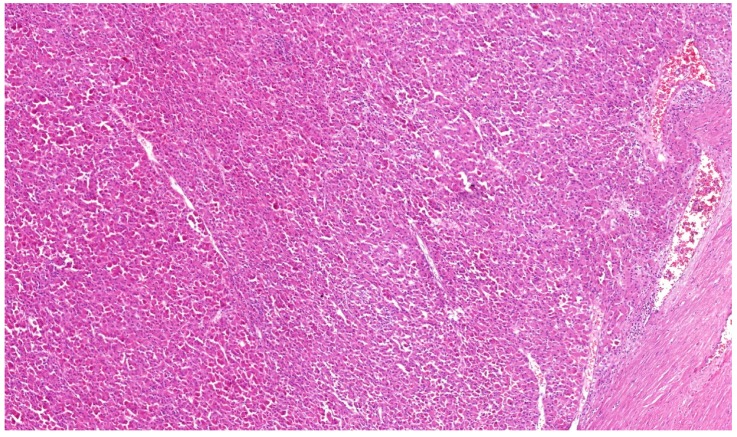
Some renal cell carcinomas (RCCs) with *TFEB* translocation lack typical morphology with pseudorosettes and rather show solid architecture. 4× magnification.

*TFEB* or t(6;11) translocation RCC is much less common member of the MiT family RCCs. These tumors exhibit a typical biphasic morphologic feature composed of large epithelioid cells with clear/eosinophilic cytoplasm and a minor population of small eosinophilic cells that form rosette-like structures within basement membrane-like material. Immunohistochemically, these neoplasms express melanocytic markers (HMB45 and/or Melan A). Usually there is a fusion of *MALAT1* and *TFEB*, although other partners such as *COL21A1*, *CADM2*, and *KHDRBS2* have recently been described [27,87]. However, even in the group of *TFEB* or t(6;11) translocation RCC, there is morphologic variability and that not all cases follow a “classic“ morphologic pattern with biphasic morphology (Figure 1).

Recent studies have shown that amplification of the *TFEB* gene in TFEB or t(6;11) translocation RCCs can uncommonly occur and is associated with more aggressive clinical behavior with distant metastases (see *RCC* with *TFEB* amplification). It is worth noting that amplification of *TFEB* gene can rarely be found in various renal tumors, most of which are usually unclassified RCCs or translocation-like RCCs.

Application in routine practice:

Diagnosis of TFE3 translocation RCC should be considered in RCC with a mixture of clear cell and papillary features, psammoma bodies, abnormally voluminous cytoplasm, hyalinized stroma, or in a young/pediatric patient. Although positive immunohistochemical staining for TFE3 or TFEB proteins, melanocytic markers, or cathepsin K can be suggestive, molecular genetic testing is highly recommended for confirming the diagnosis. FISH for *TFE3* or *TFEB* rearrangement is a helpful diagnostic tool; however NGS is recommended in cases where false negative FISH can be expected (namely partners *RBM10*, *RBMX*, *GRIPAP1*, and *NONO*). In other words, when the morphology and/or immunohistochemical profile is suggestive of TFE3 translocation RCC, NGS analysis is recommended for confirmation. Amplification of *TFEB* gene seems to be a strong adverse prognostic indicator in TFEB translocation RCCs, however such cases are rare and less frequently encountered comparing with *TFEB* amplified RCCs (without *TFEB* break) its occurrence is rather rare.

## 9. Mucinous Tubular and Spindle Cell Carcinoma (MTSCC)

Mucinous tubular and spindle cell carcinoma (MTSCC) is usually a non-aggressive renal tumor with characteristic morphology. This neoplasm can resemble PRCC with overlapping morphologic and even immunohistochemical features [88,89,90]. In the past, studies reported variable CNV patterns for MTSCC, even sometimes resembling PRCC CNV pattern suggesting MTSCC to be a variant of PRCC type 1 [36,91]. However, recent studies have shown that MTSCCs typically have a CNV pattern with multiple chromosomal losses involving chromosomes 1, 4, 6, 8, 9, 13, 14, 15, and 22, without the gains of chromosomes 7 and 17 [92,93,94,95]. In cases where there is a morphologic overlap with PRCC (mostly type 1), CNV also shows overlapping features with frequent gains of chromosomes 7 and/or 17.

Application in routine practice:

MTSCC is an indolent and rare tumor with characteristic morphologic features that can be used in diagnosis in the vast majority of cases (with or without immunohistochemical studies). In difficult cases, CNV pattern analysis can be helpful. Tumors with features of PRCC, including gain of chromosome 7 or 17, should be classified as PRCC NOS.

## 10. Tubulocystic Renal Cell Carcinoma (TC-RCC)

Tubulocystic RCC (TC-RCC) is a relative new entity first officially included in the 2012 ISUP Vancouver Classification. Similar to MTSCC, TC-RCC has morphologic and immunohistochemical features that are frequently overlap with PRCC [36,96,97].

The genetic features of these tumors are variable with previous studies suggesting similar CNV patterns to that reported in type 1 PRCC (gain of chromosome 7 or 17 and loss of Y). However, more recent studies showed that gain of chromosomes 7 and 17 is not a typical CNV pattern in cases of TC-RCC where strict histo-diagnostic criteria are applied [96,97,98]. In fact, loss of chromosome 9 has been suggested as a characteristic feature of TC-RCC [99]. It should be noted that TC-RCC is a rare and indolent tumor that should not be confused with fumarate hydratase (FH)-deficient RCC, where the tumor shows a low grade tubulocystic pattern and with abrupt transition to high-grade infiltrative carcinoma. A similar situation exists in tumors with pure tubulocystic pattern and eosinophilic cells but with prominent macronucleoli. Such cases must be considered as potentially FH deficient RCCs and immunohistochemical/molecular-genetic examination of FH should be performed [100,101].

Application in routine practice:

TC-RCC should be diagnosed based on its strict histologic criteria, without mixed areas resembling PRCC. If CNV patterns show gains of chromosome 7 and 17, it is advised to best classify it as PRCC than TC-RCC. RCCs with “Tubulocystic” features and high grade abrupt areas should raise the possibility of FH-deficient RCC and be further genetically tested for *FH* gene mutation/LOH.

## 11. Acquired Cystic Kidney Disease (ACD)-Associated Renal Cell Carcinoma

Acquired cystic kidney disease (ACD)-associated RCC is a relatively rare renal tumor. Its morphologic feature is relatively variable, as is its immunohistochemical profile. However several studies described gains of chromosomes 7 and 17, and other showed gain of chromosomes 3, 16, and Y [102,103,104,105,106,107].

Application in routine practice:

Currently there are no specific genetic alterations useful for routine practice in these tumors.

## 12. Renal Medullary Carcinoma

Renal medullary carcinoma is a rare, aggressive, and high grade renal tumor occurring mostly in African Americans with sickle cell trait or with other hemoglobinopathies. Within the differential diagnosis, collecting duct carcinoma, high-grade urothelial carcinoma and other high-grade RCCs should be always considered. [108] Medullary carcinoma is characterized by loss of the *SMARCB1* (INI-1) gene [109,110,111], which can also be detected immunohistochamically (following by positive OCT3/4 staining) [108,112,113,114,115]. In rare cases where alterations of *SMARCB1* gene or abnormal negative staining for the protein is documented in the absence of sickle trait, the term “RCC unclassified with medullary phenotype” has been proposed [116,117].

Application in routine practice:

High-grade renal tumors with histologic features suggestive of renal medullary carcinoma should be stained with SMARCB1. For cases with loss of SMARCB1 expression, molecular genetic testing of *SMARCB1* is useful. The result of immunohistochemical/genetic testing should be correlated with hematologic findings (i.e., sickle cell trait or other hemoglobinopathy). In situation, when RCCs with *SMARCB1* loss is encountered, and sickle trait or other hemoglobinopathies are absent, it is currently recommended to classify them as RCC unclassified with medullary phenotype.

## 13. Collecting Duct Carcinoma (CDC)

One of the most frequently misclassified renal tumors is still collecting duct carcinoma (CDC). Even nowadays, the diagnosis of CDC remains the diagnosis of exclusion. The following entities should be always be considered and excluded in such scenarios: FH-deficient RCC, high-grade urothelial carcinoma of renal pelvis, renal medullary carcinoma, and metastatic carcinoma from another organ.

Unfortunately, currently there is no characteristic molecular genetic feature or combination of features useful for differential diagnosis. Molecular genetic testing should be considered after excluding other entities in the differential diagnosis (i.e., FH-deficient RCC, renal medullary carcinoma).

Application in routine practice:

There is no specific molecular genetic test which can help to establish the diagnosis of CDC. FH-deficient RCC and renal medullary carcinoma should be always considered and diagnosis can be supported by genetic testing.

## 14. Succinate Dehydrogenase (SDH)-Deficient Renal Cell Carcinoma

**Figure 2:**
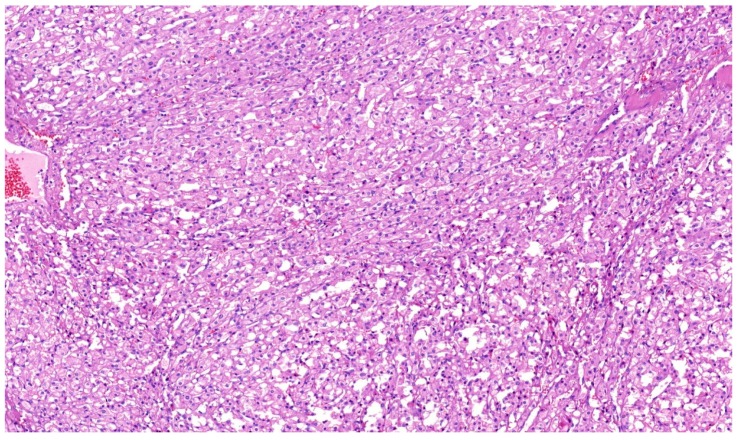
SDHB-deficient RCC with typical morphology-eosinophilic renal tumor with numerous vacuoles resembling texture of bubble wrap. 10× magnification.

Renal tumors associated with autosomal dominant germline mutations of *SDHA*, *SDHB*, *SDHC* and *SDHD* have recently been described. Such tumors are part of syndrome characterized by occurrence of renal carcinomas, paragangliomas/pheochromocytomas, gastrointestinal stromal tumors (GIST), and pituitary adenomas [118,119]. The majority of succinate dehydrogenase (SDH)-deficient RCCs demonstrate a characteristic morphology with solid alveolar architecture, eosinophilic cytoplasm with numerous intracytoplasmatic vacuoles (Figure 2). Cases with high grade features and overlapping morphology resembling CCRCC, PRCC or unclassified RCC have also been described. Immunohistochemical staining for SDHB is negative. Antibody against SDHB detects all 4 subgroups (*SDHA*, *SDHB*, *SDHC* and *SDHD)* deficiencies [118,119]. However, the interpretation of SDHB staining must be done with caution and an internal positive control should be present. SDH deficiency is almost always associated with germline SDH subunit mutation [118,119,120,121].

Application in routine practice:

Suspected cases should be immunohistochemically stained for SDHB. Immunohistochemical staining for SDHB is negative in SDH-deficient cases. The vast majority of SDH-deficient RCCs are associated with germline mutation of the *SDHB* subunit. Genetic testing of *SDH* subunit mutation is not necessary, however in cases where the result of immunohistochemical examination is inconclusive, it is highly recommended.

## 15. Fumarate Hydratase (FH)-Deficient RCC and HLRCC (Hereditary Leiomyomatosis and Renal Cell Carcinoma)

**Figure 3:**
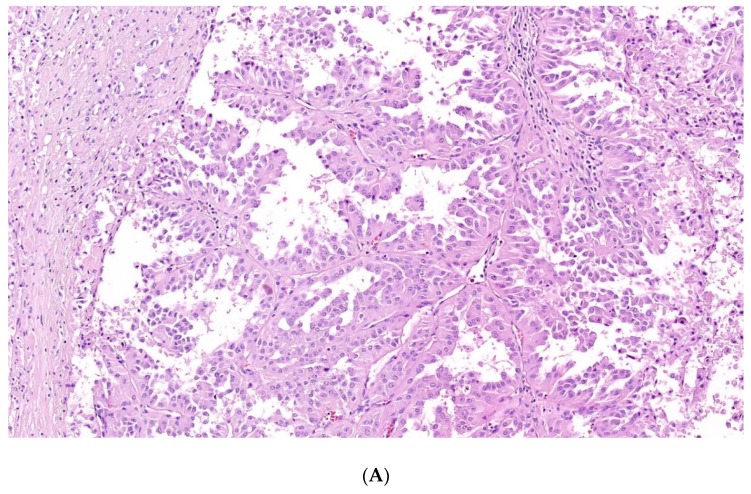

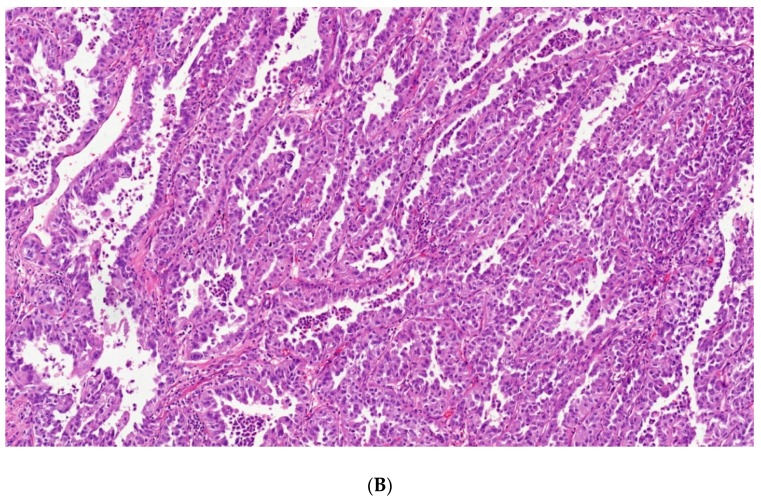
FH-deficient RCC: deep red macronucleoli can be prominent (**A**), however in some cases it is not easy to detect them (**B**). 4× magnification.

FH-deficient RCC and hereditary leiomyomatosis and renal cell carcinoma associated RCC have been discussed extensively in the recent literature. Initially it was thought that these tumors are hereditary counterparts of the so-called “type 2” PRCC. Histologically, they show marked intratumoral heterogeneity with papillary, tubulocystic, solid or cribriform patterns, and usually the presence of large nuclei with deep red nucleoli (Figure 3A,B). However, no single or a combination of histologic features are diagnostic of FH-deficient RCCs/HLRCCs [101,108,122,123,124,125,126,127,128].

Immunohistochemically, FH-deficient RCCs show loss of staining for fumarate hydratase (FH) (sensitivity 80 to 90%) [101,108,123,124,125,126,127,128,129]. Positive immunohistochemical staining for 2SC (2-Succinocysteine) is supportive feature, however antibody for 2SC is not currently commercially available [123,125,129]. The CNV pattern is heterogeneous, no constant combination of changes has been disclosed so far and it is not possible to use it in differential diagnostic process [124].

Overall, in cases with suspected clinical and morphologic features (high-grade aggressive RCCs in young patients) FH-deficient RCCs/HLRCCs should be considered in the differential diagnostic workup. For screening, immunohistochemical staining with FH is useful, however cases where staining interpretation is not convincing or in suspected clinical settings it would be better to test for *FH* mutation/LOH.

Application in routine practice:

High-grade RCCs occurring in young patients exhibiting variable growth patterns and morphologic features should prompt the differential diagnosis of FH-deficient RCCs/HLRCCs. Immunohistochemically, FH can be helpful; however, it is not 100% specific, and as such analysis of *FH* mutation/LOH should be considered.

## 16. New but Perspective Renal Tumors

As mentioned earlier in the introduction, renal tumors are intensively studied and more new entities and variants are described every year. It is questionable whether all these variants will be regarded as established entities within future classifications or whether they will be reclassified as variants of some “traditional” renal tumors. Some of the published papers are recent without further corroboration by other studies, while others worked with a limited number of cases. More studies examining the ideas and hypotheses would be needed to allow including such entities in the future WHO classifications. In the following section we will briefly introduce such tumors. Majority of new entities will be covered in other reviews in this issue of *Cancers*.

### 16.1. Eosinophilic Solid and Cystic (ESC) RCC

Eosinophilic solid and cystic RCC (ESC-RCC) is a recently recognized entity, described in patients with TS (tuberous sclerosis) complex. Subsequently, identical tumors were described in patients without any relation to TS complex, mostly middle aged/elderly women [130,131]. These tumors have solid and cystic architecture, composed of neoplastic cells with voluminous cytoplasm showing basophilic stippling [132]. They are frequently positive for cytokeratin 20, which is highly unusual for any RCCs [132]. Both familiar and sporadic tumors have molecular alterations of *TSC1* or *TSC2* [133,134,135,136,137].

### 16.2. RCC with TSC/MTOR Gene Mutations

The molecular genetic revolution in the field of oncopathology has resulted in identifying more entities including a recently described subset of tumors harboring mutations of *TSC1*, *TSC2*, or *MTOR*, being recognized in sporadic patients as well as patients with tuberous sclerosis complex [130,131]. RCC with prominent smooth muscle (or sometimes referred as RCC with angioleiomyoma-like stroma), [130,138,139,140,141,142], tumors with oncocytic features named as HOT (high-grade oncocytic tumor) or descriptively as sporadic RCC with eosinophilic and vacuolated cytoplasm [143,144,145] are best known examples of this group.

### 16.3. TCEB1-Mutated RCC

These tumors are well-circumscribed, have predominantly tubular and papillary architecture, and have thick intersecting fibromuscular bands superficially resembling a renal angiomyoadenomatous tumor (RAT)-like morphology. They are distinct from both CCRCC and CCPRCC, harboring mutations of *TCEB1* but with no *VHL* gene abnormalities [24,138,140,146,147,148]. Given the limited data available on these tumors, it is rather early to assume concrete conclusions [146].

### 16.4. RCC with TFEB/6p21/VEGFA Amplification

**Figure 4:**
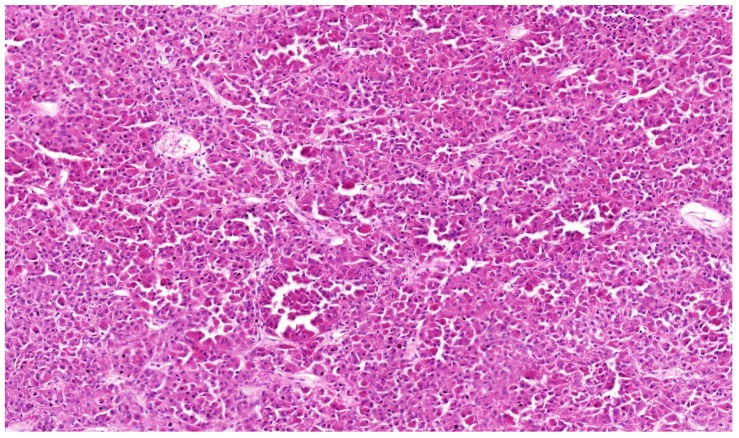
RCC with *TFEB* break and *TFEB* amplification. 4× magnification.

RCC with *TFEB* rearrangement is a poorly understood entity, although such tumors have been described or briefly mentioned in several papers. The first systematic study summarizing knowledge about this group of tumors was published by Williamson at al. [149] in 2017. It appears that tumors from this group show amplification of chromosome 6p21 with changes in *TFEB* and *VEGFA* [149,150,151,152,153,154,155,156]. So far the described cases show variable morphology with shared positivity for melan-A and/or HMB45. Cathepsin K is usually positive [149,150]. *RCC* with *TFEB*/*6p21*/*VEGFA* amplification exhibit papillary architecture, however tumors resembling CCRCC or ChRCC were also documented. Molecular genetics usually disclose amplification of *TFEB*/*6p21*/*VEGFA*, while rearrangement of *TFEB* is usually not present. However, in one of the first cases authors pointed out that amplification of TFEB gene might be a marker of aggressive behavior showed both rearrangement and amplification [152] (Figure 4). Recent work shows that *TFEB* gene expression is increased in these tumors, although not as much as in *TFEB* translocation tumors, raising the possibility that other genes at the 6p21 locus, such as *VEGFA* or *CCND3* or other genes may be responsible for aggressive behavior [155].

### 16.5. ALK-Rearranged RCC

Rearrangement of *ALK* has been described in various tumors, mostly in lymphomas, lung carcinomas, and thyroid carcinomas. In kidney, renal tumors with *ALK* rearrangement have also been rarely reported [157,158,159,160,161,162,163,164,165,166,167,168,169,170,171,172,173,174]. Histologically, they show a tubulopapillary or cribriform pattern with rhabdoid-like cell morphology in a myxoid/mucinous background (mostly interstitium). Fusion partners that have been identified in *ALK*-rearranged RCC are *TPM3*, *STRN*, *VCL*, *HOOK1*, *CLIP1*, and *KIF5B.* Some cases demonstrated highly surprising morphology, identical to metanephric adenoma or MTSCC [166].

## 17. Discussion

It is well-known that renal tumors are characterized by marked both intertumoral and intratumoral heterogeneity, which can play role in tumor evolution and hamper personalized therapeutic strategies. Molecular characterization of renal cell neoplasms has led to the identification of driver genes and specific molecular pathways. This comprehension along with the traditional histo-morphologic features has revolutionized the treatment approach and modalities in these tumors.

Imaging genomics, an emerging research field, has also created new opportunities for the diagnosis and prognosis of renal tumors. Of note, Cheng et al. [175] developed and examined an integrative genomics framework for constructing a prognostic model for clear cell renal cell carcinomas using both histopathologic images and genomic signatures. Similarly, Shao et al. [176] introduced ordinal multi-modal feature selection framework that simultaneously identified important features from both pathological images and multi-modal genomic data for the prognosis. It appears that such an integrative pathologic-genomics approach can help to better understand prognostic and hopefully therapeutic aspects of various renal tumors.

It should be noted that one of the main challenges in assessing the current literature on molecular-genetic characteristics of renal tumor is related to the heterogeneity of methodologies and definitions used in various studies. This is mainly due to the fact that our understating of renal neoplasms is evolving as the new molecular and technological advances are emerging such as NGS. Despite the limitations of the current literature, we are still able to draw the landscape of uniform histo-molecular renal entities.

## 18. Conclusions

Overall, most renal tumors can easily be diagnosed based on pure histologic findings with or without immunohistochemical examination. However, in selected cases, molecular-genetic testing can be utilized to assist in arriving at an accurate diagnosis.

## Figures and Tables

**Table 1:** Genetic tests and routinely used immunohistochemical examinations in renal cell tumors

Tumor Type	IHC	Mutation	Method
CCRCC	Carbonic Anhydrase (CA) IX, Vimentin	VHL (von Hippel Lindau) inactivation	Sequencing (NGS/classical)/methylation specific PCR (polymerase chain reaction)
MCRCNLMP	NS	VHL	NR
PRCC type 1 hereditary syndrome	CK7, AMACR	*MET*	Sequencing (NGS/classical)
PRCC type 1 conventional type	CK7, AMACR	Gain of 7,17	aCGH/FISH
PRCC type 2	NS	NS	
OPRCC	AMACR, Vimentin	KRAS	Sequencing (NGS/classical)
FH-deficient RCC	FH, 2SC	*FH* mutation/LOH analysis	Sequencing (NGS/classical)/fragment analysis
ChRCCC	CK7, CD117	NS	
“Hybrid” On/Ch tumors	NS	FLCN **	Sequencing (NGS/classical)
Oncocytoma	CK7, CD117	NS	
Clear cell PRCC	CK7, AMACR	VHL	Sequencing (NGS/classical)
MiT RCC	TFE3, Cathepsin K	TFE3, TFEB	FISH/NGS
MTSCC	AMACR, EMA	CNV pattern analysis	aCGH
TC-RCC	CK7, AMACR	CNV pattern analysis	aCGH
ACD-associated RCC	CK7, AMACR	NS	
RMC	INI1	*SMARCB1*	Sequencing (NGS/classical)
CDC	34betaE12, Ck7	NS	
SDH-deficient renal cell carcinoma	SDHB	SDHB	Sequencing (NGS, classical)/IHC

Clear cell renal cell carcinoma (CCRCC). Multilocular cystic renal cell neoplasm of low malignant potential (MCRCNLMP). Papillary RCC (PRCC). Oncocytic papillary RCC (OPRCC). “Hybrid” oncocytic/chromophobe tumors (Hybrid” On/Ch tumors). ** Diagnosis of Birt–Hogg–Dubé syndrome. (MTSCC) Mucinous tubular and spindle cell carcinoma. CNV (copy-number variation) Tubulocystic RCC (TC-RCC) Renal medullary carcinoma (RMC). Fluorescence in situ hybridization (FISH). Next-generation sequencing (NGS). Array comparative genome hybridization (aCGH). Not specific (NS). Immunohistochemistry (IHC). Not recommended (NR).
